# Reply to: The influences of the environment and information on the complications of diabetes on patient outcomes

**DOI:** 10.1186/s12955-021-01831-8

**Published:** 2021-08-24

**Authors:** Zeynep Bahadır Ağce, Gamze Ekici

**Affiliations:** 1 Department of Occupational Therapy, Uskudar University, İstanbul, Turkey; 2 Department of Occupational Therapy, Hacettepe University, Ankara, Turkey

We read with great interest this study, which clarified the effects of problem-solving therapy (PST) on the occupational performance, self-efficacy, and well-being of type diabetic patients in Turkey. However, we have two concerns about the methodology of this study.

First, the difference between the two groups in terms of whether they were in an environment with or without occupation might have significantly affected the results, so the results should be subtracted. In this study, although the participants were randomly assigned at Baseline, more participants in the intervention group than in the control group were engaged in work. Therefore, the Canadian Occupational Performance Measure (COPM) for primary outcomes is more likely to have a stronger effect on the amount of change because it affects the client’s environment (2). The COPM is a questionnaire that asks about self-perceptions of occupational performance. Participants in the environment with occupation might find it easier to visualize and practice “problem definition,” “generation of alternatives,” “decision-making,” “solution implementation and 16 verification,” in PST. 17.

- Two concerns have been expressed about the methodology of the study entitled "Person-Centred, Occupational-Based Intervention Program Supported by Problem Solving Therapy for Type 2 Diabetes".
  The first concern was the difference between the two groups in terms of whether they were in an environment with or without occupation might have significantly affected the results. It has been suggested by the reviewer that the participants in the environment with occupation might find it easier to visualize and practice “problem definition,” “generation of alternatives,” “decision-making,” “solution implementation and verification,” in PST. In this study, regression analysis was used to evaluate the effect of demographic variables including gender, occupational status, diabetes duration, treatment regime on the results. Univariate and multivariate regression analysis results in both the control and intervention groups were significant only for ineffective and effective coping change scores (p < 0.05). Regression coefficients showed that effective coping scores were more effective than the ineffective coping scores in both the control and intervention groups. Effect sizes were found to be higher for both ineffective and effective coping scores in the control group. In addition, it was also determined that in identifying and prioritizing important activities, the ineffective coping style was found more effective in the univariate regression, while the effective coping style in the multiple regression analysis. These results confirmed that different working conditions of the individuals did not cause a difference between the groups. Therefore, we are of the opinion that the claim that the participants in the environment with occupation might find it easier to visualize and practice “problem definition,” “generation of alternatives,” “decision-making,” “solution implementation and verification,” in PST is not valid.

Second, this study does not describe the complications and treatment of diabetes, which we would like to know about. Hypoglycemia, a complication of diabetes, is said to reduce the work productivity of diabetic patients. In addition, patients with type 2 diabetes are limited by the social and emotional aspects of insulin therapy [4]; they refuse insulin therapy because they feel that starting insulin will stigmatize them socially. Thus, depending on the complications and treatment of diabetes, the results of this study may be weakened.

- The second concern is that the study did not describe the complications and treatment of diabetes, thus it has been suggested that the results of this study may be weakened depending on the complications and treatment of diabetes. Diabetes treatment and complications were not directly described in the study. However, the Canadian Occupational Performance Measure (COPM) was used to express diabetic self-care and occupational performance problems related to diabetes complications. The COPM is based on a semi-structured interview method and helps individuals to identify, prioritize, and evaluate important issues they encounter in the defined occupational performance areas. The use of COPM has made it possible to identify performance problems in different areas that may arise due to diabetes complications or treatment, and therefore the solution process. For example, among the occupational performance problems shared in the total score under the heading of "personal care" in Table 3, the problems such as monitoring, foot care, smoking, wearing socks, bathing were defined. These occupational problems have been directly or indirectly related to the treatment or complications (Table 3 can be presented in more detail upon request). Problem-solving therapy has been a supportive approach in overcoming the occupational performance problems that develop due to diabetes treatment and complications.

I hope I clarified your questions, ıf you need more information you can reach me by fztzeynepbahadir@gmail.com.

## Abbreviations

PST: Problem-solving therapy
COPM: The Canadian Occupational Performance Measure
